# Do children and adolescents have a future-oriented bias? A developmental study of spontaneous and cued past and future thinking

**DOI:** 10.1007/s00426-018-1077-5

**Published:** 2018-08-25

**Authors:** Teresa McCormack, Patrick Burns, Patrick O’Connor, Agnieszka Jaroslawska, Eugene M. Caruso

**Affiliations:** 1 School of Psychology, Queen’s University Belfast, Belfast, BT71NN UK; 2 School of Philosophy, Psychology, and Language Sciences, University of Edinburgh, Edinburgh, UK; 3 UCLA Anderson School of Management, Los Angeles, USA

## Abstract

Previous research has indicated that adults have a future-oriented cognitive bias, one illustration of which is their tendency to report more thoughts about the future than the past during mind-wandering. We examined whether children showed a similar bias, and whether there were any developmental changes in the magnitude of such a bias. Children aged 6–7 and 9–10 years, adolescents, and adults completed two tasks in which they could report either past or future thoughts: a mind-wandering task assessing spontaneous past and future thinking and a cued episodic thinking task in which they were free to describe either past or future events. Only adults showed a future-oriented bias in the mind-wandering task. Participants in all groups were much more likely to describe past events in the cue word task, and the proportion of future events described did not change developmentally. However, more than a third of the youngest age group produced no descriptions at all of future events, which was a significantly larger proportion than in any other age groups, and illustrates the difficulty that some children of this age have with future thinking. Our findings indicate that future-oriented bias and developmental changes in such bias may be task-specific.

## Introduction

Research on thinking in adults has suggested that there is what has been termed a “future-oriented” or “prospective” bias (Grant & Walsh, 2016; Smallwood, Nind, & O’Connor, 2009). Experience sampling techniques in the laboratory and in the context of daily life have indicated that adults typically report future-oriented thoughts more frequently than past-oriented thoughts (e.g., Grant & Walsh, 2016; Smallwood et al., 2009; Smallwood et al., 2011; Spronken, Holland, Figner, & Dijksterhuis, 2016; Stawarczyk, Cassol, & D’Argembeau, 2013; Stawarczyk, Majerus, Maj, Van der Linden, & D’Argembeau, 2011; Song & Wang, 2012). Adults attach greater value to future events than past events, feel greater emotions when considering future events compared to past events, and judge the future to feel closer than the past (Caouette, Wohl, & Peetz, 2012; Caruso, 2010, Caruso, Gilbert, & Wilson, 2008; Caruso, Van Boven, Chin, & Ward, 2013; Van Boven & Ashworth, 2007); such findings can also be interpreted as evidence of a future-oriented bias in judgments and attitudes (Suhler & Callender, 2012; Sullivan, 2018).

Numerous authors have emphasized the adaptive nature of future-oriented cognition in allowing us to prepare for a future that is as yet undetermined, in comparison to merely dwelling on past events that cannot be changed (e.g., Parfit, 1984; Schacter, Addis, & Buckner, 2007; Suddendorf & Corballis, 2007; Suhler & Callender, 2012). On such an approach, a future-oriented bias can be seen as an evolutionary adaptation. What is not clear, though, is the developmental origins of such a bias (though see Burns, McCormack, Jaroslawska, Fitzpatrick, McGourty, & Caruso, 2018). In particular, very little is known about the salience of the future in the mental lives of children. Children begin to use and understand the future tense in language at the early stages of language acquisition (Harner, 1982; Weist, 1989), and research on the development of episodic future thinking suggests that when prompted, children can reliably begin to describe specific future events around 3–4 years of age (Busby & Suddendorf, 2005; Hayne, Gross, McNamee, Fitzgibbon, & Tustin, 2011; Quon & Atance, 2010; Richmond & Pan, 2013). However, although it has been suggested that episodic future and past thinking develop in tandem in the preschool years (Busby & Suddendorf, 2005; Richmond & Pan, 2013), studies of middle childhood indicate that the ability to episodically remember the past develops more quickly than the ability to episodically imagine the future (Coughlin, Lyons, & Ghetti, 2014; Wang, Capous, Koh, & Hou, 2014). If children do have particular difficulty episodically imaging the future, this might translate into future thoughts playing *less* of a role in their mental lives.

The aims of the current study are to examine whether children show future-oriented bias, as operationalized by a tendency to think about the future rather than the past, and whether there are developmental changes in the magnitude of such a bias across childhood, adolescence, and into adulthood. We measured the tendency to think about the future versus the past in two ways: using a mind-wandering task and a word-cueing task that prompted episodic thinking.

### Mind-wandering

Adult studies have examined spontaneous future and past thoughts using experience sampling methods, and in particular by looking at the temporal focus of mind-wandering in laboratory-based testing (e.g., Baird, Smallwood, & Schooler, 2011; Smallwood et al., 2009, 2011). Given the educational significance of mind-wandering (Smallwood, Fishman, & Schooler, 2007), it is perhaps surprising that there are extremely few developmental studies, and no existing data that we are aware of that examine whether there are developmental changes in the temporal focus of mind-wandering. In a recent developmental study, Van den Driessche et al. (2017) examined mind-wandering (and relatedly, “mind-blanking”) in children of a mean age of 8 years using a paradigm similar to that typically used with adults. The focus of their study was actually on the effects of ADHD, and they did not examine developmental change and also did not report the temporal focus of mind-wandering in their child samples. Stawarczyk, Majerus, Catale, and D’Argembeau (2014) compared levels of mind-wandering in adolescents and adults but did not ask participants to report whether mind-wandering was to the past or future. Two earlier studies of mind-wandering in late childhood, however, did report temporal focus. Zhang, Song, Ye, and Wang (2015) examined mind-wandering in 9- to 11-year-olds who were performing a Go-NoGo task and found that children reported more future-oriented episodes of mind-wandering than past-oriented episodes. In Ye, Song, Zhang, and Wang’s (2014) study, children aged 9–14 (mean age of 11 years) reported on mind-wandering while performing a choice reaction time task or a 1-back working memory task. For both tasks, children reported thinking about the future around 25% of the time, whereas they reporting thinking about the past 11–14% of the time. The findings of both Ye, Song, Zhang, & Wang (2014) and Zhang et al.’s (2015) studies suggest that by late childhood, a future-oriented bias is apparent. However, developmental changes have not been examined, and it is not known if such a bias exists in younger children.

A number of findings suggest the hypothesis that the temporal focus of mind-wandering may shift developmentally towards becoming more future-biased. First, developmental changes in executive functioning, which are well-documented to occur over a long developmental period (Best & Miller, 2010), might result in changes in temporal focus. Baird et al. (2011) found that adults with higher working memory capacity showed a greater future-oriented bias in mind-wandering (though see McVay, Unsworth, McMillan, & Kane, 2013; Robison & Unsworth, 2017); moreover, increasing the demands of the primary task on working memory capacity reduces the future-oriented bias (Smallwood et al., 2009, 2011). Both of these findings suggest that the future-oriented bias in mind-wandering may be linked to available working memory capacity, which in turn suggests that as working memory capacity increases, a future-oriented bias may emerge or become more pronounced.

Second, there is some evidence that brain regions which are known to develop across childhood and into adolescence (Blakemore & Choudhury, 2006; Hooper, Luciana, Conklin, Yarger, 2004) are critical for the future-oriented bias in mind-wandering. Bertossi and Ciaramelli (2016) reported that patients with ventromedial prefrontal damage not only showed reduced overall levels of mind-wandering, but most strikingly, reported no future-oriented mind-wandering at all. These authors argue that this pattern stems from impairments in episodic thinking (Bertossi, Tesini, Cappelli, & Ciaramelli, 2016) which may be particularly pronounced for episodic future thinking (Bertossi, Candela, Ciaramelli, & De Luca, 2017). Although it is difficult to straightforwardly extrapolate developmental predictions from studies of neuropsychological patients, these findings indicate that the future-oriented bias in mind-wandering may depend on brain regions and associated cognitive functions that develop gradually.

In our study, we used a simplified mind-wandering paradigm in which participants reported when probed as to whether they were thinking about the past, present, or future while they completed another task. Because we were concerned about young children’s potential difficulties in reporting on mind-wandering while simultaneously completing a cognitively demanding task, and because we wanted to match task difficulty as closely as possible across age groups, we asked participants to do some coloring-in rather than the more standard type of cognitive task typically used in mind-wandering studies with adults. They were then probed at intervals to report, by pressing one of three buttons on a touch-screen, whether they were thinking about the past, the here-and-now, or the future. We note that the “here-and-now” category of response does not distinguish between whether children were thinking about the coloring-in task or other aspects of their present environment. However, we wanted to keep the response options simple for young children, and our primary focus was on the relative proportions of past versus future responses.

### Cued episodic thinking

Our participants also completed an episodic thinking task using a cue word technique. Cue word studies of episodic thinking and its development have been conducted previously (e.g., Coughlin et al., 2014; Quon & Atance, 2010); what was distinctive about our task was that participants were free to generate either a past or a future episode in response to the cue, whereas in previous studies, participants were explicitly directed to describe only either past or future events. We were interested in the relative proportions of past and future episodes that participants generated in response to the cues and specifically if these proportions changed developmentally. In addition, we asked for participants to make ratings about the clarity of the events in their minds, how quickly the event came to mind, and the valence of the event. For these additional measures, we were interested in past–future differences, and also whether these showed a developmental pattern.

To the best of our knowledge, this was the first study to examine cued episodic thinking in which participants were free to report either past or future event descriptions. However, there are some existing findings that make it possible to speculate on the likely temporal focus, and on past–future differences regarding the additional measures. With regard to the former issue, it might be predicted that temporal focus would not show future-oriented bias even in adults. In the context of mind-wandering, thoughts that are cued by environmental stimuli tend to be past- rather than future-oriented. Maillet and Schacter (2016) found that past rather than future thinking was more prevalent for episodes of mind-wandering that were triggered by presented stimuli, whereas this was not the case for thoughts unrelated to the stimuli. Similarly, Plimpton, Patel, and Kvavilashvili (2015) reported that mind-wandering was dominated by past-oriented thoughts under circumstances in which unrelated verbal cues appeared the screen (see also Vannucci, Pelagatti, & Marchetti, 2017). All these authors suggest that verbal cueing is more likely to trigger memory retrieval than future thinking. Studies of involuntary mental time travel, in which participants report when mental time travel to either the past or future occurs spontaneously also indicate the absence of a future-oriented bias; in these studies, similar amounts of past and future thought occur (Berntsen, Rubin, & Salgado, 2015; Cole, Staugaard, & Berntsen, 2016; Finnbogadóttir & Berntsen, 2013).

Although our task was not a mind-wandering task, because participants were explicitly asked to generate event descriptions in response to word cues, these findings suggest that we are unlikely to find a future-oriented bias. Nevertheless, we thought it was possible that there would be a developmental shift in the relative proportions of past versus future event descriptions, with an age-related increase in future event descriptions. Such a prediction could be generated on the basis of previous findings indicating that children find episodic future thinking particularly difficult (Coughlin et al., 2014; Wang et al., 2014). It would be consistent with suggestions that episodic future thinking is more cognitively effortful than episodically remembering because of its greater demands on constructive processes (Addis, Wong, & Schacter, 2007; D’Argembeau, Ortoleva, Jumentier, & van der Linden, 2010; Schacter & Addis, 2007; though see Anderson, Dewhurst, & Nash, 2012; Jeunehomme & D’Argembeau, 2016). Thus, we speculated that the proportion of future events described might increase developmentally.

On the basis of previous findings, we were also able to make predictions about the other measures used in our task (clarity, how quickly the event came to mind, and valence). Studies with adults suggest that the rated clarity of remembered events might be greater than imagined future events (Berntsen & Bohn, 2010; D’Argembeau & Van der Linden, 2004, D’Argembeau & Van der Linden, 2006; Gamboz, Brandimonte, & De Vito, 2010), and one might predict that such a past–future difference may be even more marked in children (although we note that Coughlin et al. 2014, did not report past–future differences in clarity in any of their age groups). In terms of self-reported speed at which the event came to mind, Coughlin et al. found that child groups reported that future events took longer to come to mind than past events, but this was not true for their adult group. Coughlin et al. speculate that children’s self-reports on this measure reflect their greater difficulties with generating future event descriptions compared to past event descriptions, whereas adults do not struggle with future event generation to the same extent. We were interested in whether we could replicate Coughlin et al.’s developmental findings regarding self-reported speed at which an event came to mind in the context in which participants could freely choose to describe a past or a future event. One possibility is that past–future differences in speed may disappear even in children under circumstances in which participants can describe whichever type of event comes to mind first.

Finally, there is considerable evidence that suggests that imagined future events are more likely to be rated as having positive valence than remembered events, both in situations in which thought about events at other times is cued and when it occurs spontaneously (Berntsen & Bohn, 2010; Berntsen & Jacobsen, 2008; Cole et al., 2016; Finnbogadóttir & Berntsen, 2013), i.e., that there is a marked future positivity bias (Cole et al., 2016). This is in line with a variety of findings that suggest that people have a tendency to assume that their future will be rosy (e.g., Newby-Clark & Ross, 2003; Ross & Newby-Clark, 1998). There is some evidence to suggest that even relatively young children tend to be (over) optimistic about their future over long-time scales (Bohn & Berntsen, 2013; Lockhart, Chang, & Story, 2002), and in their developmental study of episodic thinking. Abram, Picard, Navarro, and Piolino (2014) found a future positivity bias that did not interact with age. Thus, we anticipated that the future positivity bias might be present in all of our age groups.

### The current study

In the study to be described, we tested a notably wide age range of participants. There were two groups of children (aged 6–7 and 9–10), alongside an adolescent group (aged 14–15) and an adult group. Use of these age groups allowed us to examine the development profile of spontaneous and cued past and future thinking in detail. In addition to the mind-wandering and word-cue tasks, participants completed a task assessing working memory capacity. The latter task was included because of previous suggestions that future-oriented thinking in mind-wandering may make more demands on working memory resources than past-oriented thinking (Baird et al., 2011) and that episodic future thinking may be more cognitively demanding than episodic remembering (Addis et al., 2007; D’Argembeau et al., 2010). This allowed us to examine the role of working memory abilities in explaining any individual or group differences in future-oriented bias.

## Method

### Participants

There were four groups of participants in the study: forty two 6- to 7-year-olds (*M*_age_ = 84 months; range = 69–96 months, 24 females), thirty one 9- to 10-year-olds (*M*_age_ = 120 months; range = 108–139 months, 10 females), twenty three 14- to 15-year-olds (*M*_age_ = 14 years 3 months; range = 153–188 months, 12 females) and thirty-seven adults (*M*_age_ = 27 years 5 months; range = 18–63 years, 25 females). All participants were tested in the developmental laboratory in the home department of the first author. Two participants from the youngest age group did not contribute data to the word cue task due to equipment error and experimenter error, respectively. One further participant from the youngest age group refused to give any responses during the digit span task. One participant each from the 14- to 15-year-olds, and adult age groups did not contribute data to the mind-wandering data task due to equipment failure. Adult participants were primarily recruited through notices across the university, and were paid £15 (UK) for their participation. Child and adolescent participants were primarily recruited by advertising in social media and through information distributed in schools.

## Materials

**Fig. 1:**
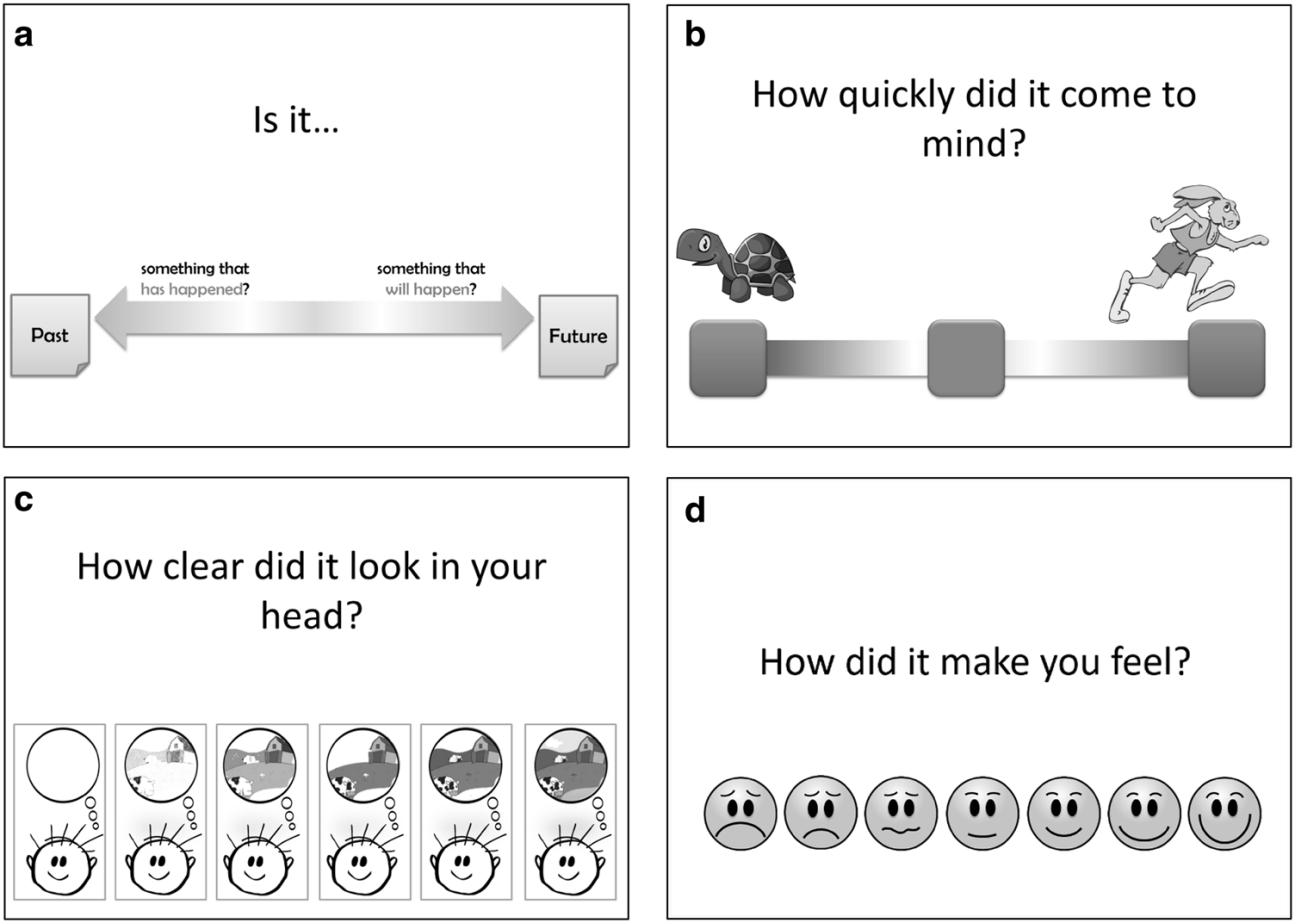
**a**–**d** Temporal focus, speed, clarity and emotion test questions as presented to participants in the word cue task

A touch-screen Dell Latitude laptop with a 12-inch screen was used to administer the tasks. In the word cue task, there were four different displays (see Fig. 1a-d). The first display (Fig. 1a) had two squares on the screen, one on the left for reporting past events (also described as something that has happened) and one on the right for reporting future events (described as something that will happen). The second display (Fig. 1b) was used to report how quickly the event came to mind using a three-point scale ranging from slow to fast. The square on the left had a picture of a tortoise beside it and the square on the right had a picture of a sprinting hare, representing “slow” and “fast”, respectively. The middle square had no picture beside it and was used to indicate that the event came to mind neither fast nor slow. The third display (Fig. 1c) was used to report how clearly participants imagined the event, and had six pictures of a character with a thought bubble. A picture of a scene in the bubble varied systematically in how blurry it was. Finally, the fourth display (Fig. 1d) contained a horizontal row of seven faces varying from very sad to very happy to allow the participants to judge the valence of the event.

For the coloring-in task used in the mind-wandering paradigm, black-and-white printed sheets with a variety of pictures on them were used, along with a set of coloring pencils. The digit span task was taken from the WISC IV (Wechsler, 2004); we used this version even with the adult sample because we wanted to be able to look at the relation between raw scores and performance on the other tasks (e.g., to examine whether age effects might be explicable in terms of WM) and we would have had to use standardized rather than raw scores if adults had been given a different version of the digit span task. We note that adults were not at ceiling in the task (and indeed their overall mean score was slightly lower than that of the adolescents). A digital audio recorder was used to record responses in the word cue task.

### Procedure

Participants completed the three tasks in this study as part of a longer testing session lasting around 80 min. Participants completed three tasks in the following order: the word cue task, the mind-wandering task, and the digit span task. In between completing the word cue task and the mind-wandering task, participants completed another exercise that is not reported here in which they made speeded judgments about whether sentences were past or future tensed; this task took around 10 min.

### Word cue task

Child participants but not adolescents or adults participated in an initial training involving the emotion scale; this was due to previous work in our lab indicating that in the absence of such training, children primarily use the extreme ends of emotion scales. Children were introduced to the scale and the faces on the scale were described as varying from “really really sad” to “really really happy”. Children were initially asked to point to the face that matched each of seven descriptions. They were then given a set of five cards depicting events of differing valence (e.g., being caught in the rain without a coat, winning a prize), and asked to try to match them to specific faces. This was to encourage them to use faces that were not at the ends of the scale. After completing the pre-training, participants began the word cue task.

Participants were introduced to the word cue task by the experimenter explaining that they would see some words on a screen and that each time they saw a word they had to think about something that had already happened or something that was going to happen that was related to that word. The experimenter then gave two examples and also demonstrated the ratings that had to be made. The word “ball” came on the screen, and the experimenter described an event s/he had participated in that involved playing in a netball competition and losing a game when the other team scored late in the game. This event was described as “a bit annoying” by the experimenter, because valence was one of the event properties to be reported. The experimenter then said “This is something that has already happened, so I need to touch the Past button.” Getting participants to explicitly report whether the event was in the past or future functioned to prepare them for making similar judgments in the mind-wandering task. The experimenter then showed participants the speed of retrieval scale and explained it to them as a way of reporting how quickly something came to mind. The experimenter selected the tortoise button to match the speed at which the netball event was retrieved. The clarity scale appeared on the next display, and the experimenter explained that this could be used to report how clearly the event looked in your head, selecting the fourth picture on the scale for the netball event. Finally, the emotion scale appeared, and the experimenter chose the third face from the left (“a bit sad”). After this initial demonstration, a second word appeared on the screen, the word “cake”. The experimenter then described an event that was going to happen next week, which was a party for his/her mother for which s/he was going to bake her favorite chocolate cake, and then moved through the screens reporting on this event, pressing the “future” button on the first display. Before moving on to the task itself, descriptions of each of the scales were reiterated.

Following this introduction and demonstration, participants completed the task themselves. In each of ten trials, a cue word appeared on the screen (the experimenter read the word aloud for children) and participants described either an event that had happened or that was going to happen “just one time”. If participants described a repeated or ongoing event, they were reminded that the experimenter wanted them to talk about an event that had happened or was going to happen just one time. Participants then made the speed, clarity, and emotion judgments for each event before moving on to the next cue. Cue words were all nouns; the words that were selected all had neutral or mildly positive emotional significance, an age of acquisition rating between one and four years, and were of high frequency in the British National Corpus (https://corpus.byu.edu/bnc/). The cues were presented in a randomized order and included the following words: book, game, pet, song, friend, bicycle, family, rain, winter, and park.

### Mind-wandering task

**Fig. 2:**
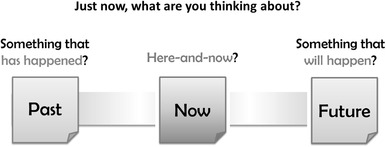
Test question as presented to participants in the mind-wandering task

The instructions for the task were displayed in a series of displays as narrated by a cartoon owl. Participants were told that they were going to do some coloring-in, and that every now and again a question would appear on the screen and they would hear a “ding” sound. They were told that the question would ask them what they were thinking about, and shown an example of the question screen, which contained three boxes arranged horizontally, with a box for “past” on the left, “now” in the middle, and “future” on the right (see Fig. 2). The experimenter explained that when they heard the ding, they would have to choose one of the boxes to report what they were thinking about, although they would not actually have to describe the contents of their thoughts. The experimenter then demonstrated the task by starting to color in. After 45 s s/he was prompted by the question, stated that s/he was thinking about buying a pair of red shoes recently, and pressed the “past” button; after a further 45 s s/he was prompted again, briefly described thinking about a future event of going for a walk and deciding whether to take an umbrella, and then pressed the “future” button. Participants then began the task themselves; the experimenter moved to a different part of the lab to leave participants seated alone at the table. Participants colored in for 12 min and during this time they were prompted six times (once every 2 min) to report on what they were thinking about.

### Digit span

Participants first completed the forward digit span followed by the backward digit span. In the forward digit span, participants were asked to listen to and repeat a sequence of numbers read aloud by the experimenter. In the backward digit span, participants were asked to repeat each sequence in reverse order. Performance was recorded on paper scoring sheets in real-time by the experimenter. Testing was discontinued after two incorrect responses to stimuli of the same length.

## Results

### Word cue task

For the purpose of analysis, responses on each of the scales were coded as follows: speed of retrieval was coded from 1 to 3, with 1 = slow, 2 = in between, and 3 = fast; clarity was coded from 1 to 6, with 1 = completely unclear and 6 = very clear; emotion was coded 1–7, with 1 = really really sad and 7 = really really happy.

Twelve participants did not give a response to all 10 cue words: 8 of the 6- to 7-year-olds (20%), 3 of the 9- to 10-year-olds (10%), and 1 of the adolescents (4%). The majority (8/12) of these participants responded to 9 out of 10 cues, and the lowest number of cues responded to was 6. Only data for the cues for which these participants produced a response were included in the analyses.

**Fig. 3:**
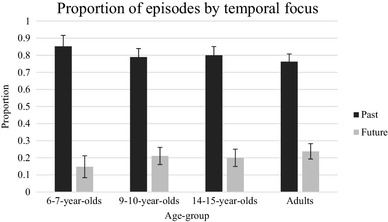
The mean proportion of responses by temporal focus in the word cue task. Error bars represent 95% confidence intervals

The first analyses examined the proportion of the described events that were from the past versus the future. Figure 3 shows these data as a function of age group. It can be seen from the figure that the majority of responses for all age groups were descriptions of past events, and the proportion of past events does not seem to vary by age. A one-way ANOVA on the proportion of events that were from the past with a between-subjects factor of age (four levels) found no significant effect of age, *F*(3, 127) = 2.26, *p* = .09, *η*_*p*_^2^ = 0.05. Because of the relatively low numbers of future events described in all groups, we also examined the proportions of participants in each group who produced no future event descriptions at all; 14 (35%) of 6- to-7-year-olds, four (13%) 9- to-10-year-olds, two (9%) 14- to-15-year-old, and 3 (8%) adults never produced descriptions of future events; chi-squared analyses showed that the proportion of participants who produced no future events varied by age group, *χ*^*2*^ (3, *N* = 131) = 12.40, *p* < .01. A greater proportion of 6- to 7-year-olds produced no future events than of either 9- to 10-year-olds, *χ*^*2*^ (1, *N* = 71) = 4.51, *p* = .03, 14- to 15-year-olds, *χ*^*2*^ (1, *N* = 63) = 5.33, *p* = .02, or adults, *χ*^*2*^ (1, *N* = 77) = 8.08, *p* < .01. There was no significant difference between the three older age groups in the proportion of participants who produced no future events (all *p* values > 0.1).

**Fig. 4:**
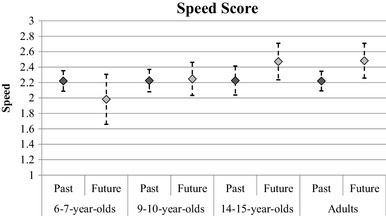
The mean speed scores across age groups for past and future events in the word cue task. Error bars represent 95% confidence intervals

**Fig. 5:**
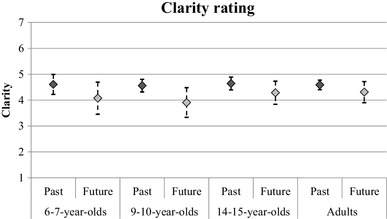
The mean clarity rating across age groups for past and future events in the word cue task. Error bars represent 95% confidence intervals

**Fig. 6:**
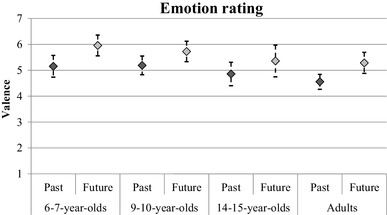
The mean emotion rating across age groups for past and future events in the word cue task. Error bars represent 95% confidence intervals

In subsequent analyses, we looked at the other measures in this task as a function of temporal focus and age group; these analyses excluded participants who did not describe any future events. Figure 4 shows the average speed of retrieval ratings for each age group and event type. A mixed ANOVA found no main effect of temporal focus, *F*(1, 104) = 1.51, *p* = .22, *η*_*p*_^2^ = 0.01, however, there was an interaction between temporal focus and age group, *F*(3, 104) = 3.95, *p* = .01, *η*_*p*_^2^ = 0.10. The interaction was explored with four paired samples *t* tests. Future episodes were judged as coming to mind quicker than past episodes in the adult group, *t*(33) = 2.79, *p* < .01. There was a marginal effect in the same direction for the adolescent group, *t*(20) = 2.03, *p* = .06. There was no difference between past and future speed estimates for either of the child groups (*p* values > 0.1). Figure 5 shows the average clarity ratings; a mixed ANOVA found a significant main effect of temporal focus only, *F*(1, 104) = 12.26, *p* < .01, *η*_*p*_^2^ = 0.11, with past events reported as being clearer in the mind (*M* = 4.60) than future events (*M* = 4.15). Finally, Fig. 6 shows the average emotion ratings; it can be seen from the figure that all age groups seem to report future events that are rated as more positively valenced than past events. ANOVA confirmed this pattern, showing a main effect of temporal focus only, *F*(1, 104) = 25.49, *p* < .01, *η*_*p*_^2^ = 0.20.

### Mind-wandering task

**Fig. 7:**
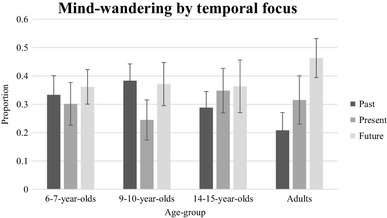
The mean proportion of mind-wandering episodes by temporal focus and age group. Error bars represent 95% confidence intervals

Figure 7 shows the proportion of times over the six probes that participants reported thinking about the past, now, or the future; two further participants (one of the 6- to 7-year-olds and one of the adults) were excluded from this analysis because they did not provide responses to all six probes. Unlike in the word cue task, few participants of any age reported no future thoughts at all (4 of the 6- to 7-year-olds, 2 of the 9- to 10-year-olds, 3 of the adolescents and 1 of the adults). In the analyses, we will classify thoughts about the past and the future as mind-wandering episodes, and assume that thoughts about the here-and-now are task-focused. A one-way ANOVA on the absolute number of times (0–6) participants reporting thinking about the here-and-now showed no significant effect of age, *F*(3, 124) = 1.04, *p* = .38, indicating that there was no developmental change in the likelihood that participants were task-focused. To examine mind-wandering, an ANOVA was then conducted on absolute numbers of thoughts with a between-subjects factor of group and a within-subjects factor of temporal focus (two levels, past or future). There was a main effect of temporal focus, *F*(1, 124) = 10.92, *p* < .01, *η*_*p*_^2^ = 0.08, with a greater number of future (*M* = 2.38) than past (*M* = 1.84) thoughts reported, and an interaction between age group and temporal focus, *F*(3, 124) = 5.19, *p* < .01, *η*^2^ = 0.11. Subsequent paired *t* tests showed that only the adult group produced more future than past thoughts, *t*(34) = 5.56, *p* < .001. One-way ANOVAs showed that the effect of age on the number of future thoughts reported did not reach significance, *F*(3, 124) = 2.54, *p* = .06, but there was a significant effect of age on the number of past thoughts reported, *F*(3, 124) = 5.30, *p* < .01, with post hoc tests showing that the adults produced significantly fewer past thoughts than either the 6- to 7-year-olds or the 9- to 10-year-olds, both *p*s < 0.05.

A final analysis examined whether there was a developmental change in the percentage of mind-wandering episodes that were to the future (i.e., the percentage of times that when participants reported thinking about other than the here-and-now, they were thinking about the future rather than the past; one participant always reported thinking about the here-and-now, and so was not included in this analysis). A one-way ANOVA found a significant effect of age, *F*(3, 125) = 6.43, *p* < .01, with post hoc Tukey tests showing that the adult group reported a higher percentage of mind-wandering episodes to the future than all of the other groups (all *p*s < 0.05), who did not differ from each other.

### Digit span

An ANOVA on scores on the digit span task with a between-subjects factor age group and a within-subjects factor of direction (forward versus backward) revealed a main effect of age, *F*(3, 128) = 24.18, *p* < .01, *η*_*p*_^2^ = 0.36, and direction, *F*(1, 128) = 189.65, *p* < .01, *η*_*p*_^2^ = 0.60, but no significant interaction between age and direction. Post hoc Tukey tests revealed that average digit span (the average of forward plus backward) of 9- to 10-year-olds was significantly greater than of 6- to 7-year-olds. Average digit span of 14- to 15-year-olds was significantly greater than of 6- to 7-year-olds and 9- to 10-year-olds. There was no significant difference between adults’ total digit span and that of 14- to 15-year-olds.

### Relationship between measures

The final analyses examined the correlations between measures. We examined, for the sample as a whole, whether there were correlations between the percentage of mind-wandering episodes that were future-oriented, the overall number of mind-wandering reports (summed across past and future), the proportion of future events described in the word cue task, and digit span performance. None of the correlations were significant (all *p* values > 0.1).

## Discussion

Our results provide some initial support for the possibility that future-oriented bias increases developmentally, but this support is not unequivocal. In the mind-wandering task, adults showed the typical pattern of reporting more future than past mind-wandering episodes, but we did not find this pattern in any of our other age groups, including the adolescents. In the word cue task, over a third of the youngest age group provided no descriptions of future events at all, a significantly higher proportion than in any other age group. However, there was no evidence of a developmental change in the average numbers of future events described in the word cue task; indeed, in all age groups including adults, the majority (around 80%) of participants’ event descriptions were of past events.

### Mind-wandering findings

Our mind-wandering task was somewhat unusual, in that, for the reasons described in the introduction, mind-wandering was assessed during a task that primarily involved motor skills rather than higher-level cognitive abilities. Under these sorts of circumstances, it would be expected that a future-oriented bias would be particularly large in adults, and indeed they reported around twice as many episodes of future-oriented mind-wandering than past-oriented mind-wandering. Strikingly, though, we did not see this pattern in any of our other groups. Although the overall number of times that participants reported future thoughts did not change developmentally, there was a developmental change in the percentage of mind-wandering episodes that were future-oriented relative to those that were past-oriented. Notably, the age pattern that we observed could not be explained by the differential availability of working memory resources in the groups; we found no relation between the percentage of mind-wandering reports that were future-oriented and working memory capacity, unlike Baird et al. (2011), but replicating the findings of McVay et al. (2013) and Robison and Unsworth (2017).

Our findings are the first to suggest that there are developmental changes in future-oriented bias in mind-wandering. However, the fact that none of the age groups other than adults showed a future-oriented bias stands in contrast to the findings of Ye et al. (2014) and Zhang et al. (2015), who both found that children of around 11 years reported more future- than past-oriented thoughts in mind-wandering. We note that these previous studies did not include an adult group, and it could be the case that the future-oriented bias they reported would have been even more marked in adults. Our task resembled that of Ye et al. (2014) in terms of the approximate length of time of the session, the number of times participants were probed regarding mind-wandering, the way mind-wandering was probed, and the use of three categories in self-report of thought content. Thus, we suspect that the difference in findings may be a result of the use of different primary tasks (choice reaction time in Ye et al.’s study versus the coloring-in task).

Such an explanation, though, is at odds with the idea that levels of future-oriented mind-wandering decrease as a function of the extent to which the primary task recruits working memory (Smallwood et al., 2009), because the main demands of our task were on motor rather than cognitive skills and thus we might have expected to see more future-oriented mind-wandering in our task compared to those of Ye et al. and Zhang et al. A more speculative explanation is that our findings reflect cultural differences between the samples (a sample from the UK versus samples from China), although we are aware of no cross-cultural evidence that would support such an explanation, and indeed it has been argued that Chinese culture is generally less rather than more future-focused than Western cultures (Guo, Ji, Spina, & Zhang, 2012). In any case, the contrast between our findings and those of Ye et al. (2014) and Zhang et al. (2015) mean that we do not want to claim that children (or adolescents) will always lack future-oriented bias during mind-wandering, and that such a bias only emerges in adulthood. Rather, the degree of such a bias might be task-dependent; our findings provide evidence that it may emerge developmentally but further studies need to examine whether this is also the case in the context of other primary tasks.

One final possible explanation of our failure to find a future-oriented bias in our child groups is that participants failed to understand the task and simply responded at random. While we did include an experimenter demonstration of the task (in response to the auditory prompt, the experimenter verbally reported two mind-wandering episodes and pressed the appropriate buttons), we did not ask participants to describe the contents of their mental states, making it impossible to be completely sure that their self-reports accurately reflected the temporal focus of their thoughts. However, we believe it is unlikely that the data could be explained by failure to understand the task. First, children had already been trained to report on the temporal focus of their thoughts (past versus future) in the word cue task, and were able to do this accurately (in the word cue task, participants did verbally describe a past or future event). This is consistent with other studies that indicate that by 6–7-year-old children can reliably report on the deictic status of events (Tillman, Marghetis, Barner, & Srinivasan, 2017). Second, it seems implausible that adolescents would fail to understand the task instructions, and this group resembled the youngest group in not showing a future-oriented bias in mind-wandering. However, to be completely confident that participants really are following task instructions, future studies could potentially ask participants to verbally describe their thoughts in response to the mind-wandering probe. We assume that studies of mind-wandering typically do not do so because it is undesirable for participants to switch their attention away from the primary task for long periods of time (thus, mind-wandering reports are designed to be brief). Nevertheless, for the purposes of checking participants’ compliance with task instructions, selected probe trials could be used.

### Word cue task: temporal focus

In the word cue task, we found no evidence of a significant developmental increase in the proportion of times participants produced descriptions of future events, although a greater proportion of the youngest children never produced any descriptions of future events than in the other groups. As inspection of the error bars in Fig. 3 shows, the youngest group produced noisier data in this task. One possibility that needs to be considered, as in the case of the mind-wandering task, is that this youngest group may have failed to understand task instructions. We can be confident that children could appropriately report on the temporal orientation of their thoughts because they provided tensed verbal descriptions. However, it is possible that some children did not realize that they could produce future as well as past event descriptions, despite the experimenter modelling the task by providing descriptions of both past and future events. We believe that this is unlikely because participants were explicitly asked to report for every event whether it was in the past or the future, meaning that they were highly likely to be aware that either option was possible. In any case, it is worth noting that the data on the temporal focus of described episodes show a similar developmental pattern (i.e., no significant change in the proportion of future events reported) regardless of whether we exclude participants from the analyses who never produced any future descriptions.

The fact that in the word cue task participants of all age groups produced descriptions of past events around 80% of the time when given a free choice between describing past and future events is consistent with the suggestion, seen in the literature on mind-wandering, that verbal cues are more likely to trigger thoughts about the past than thoughts about the future (Plimpton et al., 2015; Vannucci et al., 2017). We now consider why past event descriptions might have been so dominant in this task, even for adults.

Theorists have argued for the distinction between *direct* and *generative* modes of production for both episodic memories and episodic future thoughts, with the suggestion being that the direct mode occurs automatically and rapidly in response to a cue, whereas the generative mode is slower and involves effortful constructive processes (Anderson, Dewhurst, & Dean, 2017; Jeunehomme & D’Argembeau, 2016; Uzer & Brown, 2017; Uzer, Lee, & Brown, 2012). Recent research with adults indicates that the direct mode is responsible for around about 60% of responses in cued episodic thinking tasks, at least when the cues are object words (Jeunehomme & D’Argembeau, 2016; Uzer et al., 2012). Because, unlike in other studies, participants were free to describe either past or future events, it is plausible that they may have relied even more on the direct mode in our task. If it is assumed (at least for adults) that the majority of event descriptions result from the direct mode in the word cue task, one interesting question is why past events “win out” over future events. One possibility is that even for the direct mode past events are more accessible and retrieved more quickly than future events. However, Jeunehomme and D’Argembeau (2016) found that this was not the case, and even found some evidence that for the generative mode, descriptions of future events are produced more quickly than those of past events (although they measured retrieval speed in tasks that separately probed episodic past and future thinking). In our self-report measure, we also found that participants in all age groups did not report taking longer to bring future events to mind than past events, and indeed adolescents and adults reported that future events came to mind more quickly.

The fact that participants produced more past than future event descriptions could be seen as consistent with what Jeunehomme and D’Argembeau (2016) term the “recasting” hypothesis: that in cueing tasks assessing episodic future thinking, participants will often initially retrieve a memory of a past event, which they then simply “recast” as a description of a future episode. If this is indeed the case, then it would not be surprising to see participants producing more past than future event descriptions in our task, because the starting point for producing a future event description would often be an episodic memory of a past event: in our task participants could simply describe the past episode because there is nothing to be gained by recasting it as a future one. However, given existing findings, we believe it is unlikely that the recasting hypothesis is correct, i.e., that participants often simply re-describe a past episode as a future one (Addis, Pan, Vu, Laiser, & Schacter, 2009; Jeunehomme & D’Argembeau, 2016; though see Gamboz et al., 2010). As things stand, further studies are necessary to figure out why participants show such a marked tendency to produce past event descriptions on our task.

Although our data do not allow us to reach a conclusion as to why participants produce primarily descriptions of past events, we speculate that the proportion of past to future event descriptions may well be malleable as a function of cue type. Inspection of our own data suggests that even for specific cue words there was variability in how often future event descriptions were produced: e.g., the cue words “rain” and “bicycle” led to future event descriptions less than 10% of the time, whereas future event descriptions were given more than 20% of the time for the cue words “winter”, “family”, and “friends”. While these differences are not large, we suspect that in line with theoretical accounts of the function of episodic future thinking (Atance & O’Neill, 2001; Szpunar, Addis, McLelland, & Schacter, 2013), participants might be more likely to generate episodic future thoughts when the cues in question link with their existing goals and plans. Thus, future studies could use our paradigm and systematically manipulate the nature of the cues to examine the malleability of the tendency to produce episodic past over future event descriptions that we have reported here. Of additional interest would be whether this malleability varies developmentally; it is possible that for younger groups of children it may remain difficult to elicit episodic future thinking in such a task.

We now turn to considering the developmental findings on the other measures in the word cue task. Unlike in Coughlin et al.’s (2014) study, we did not find that children reported that future events took longer to come to mind than past events; this may reflect the nature of our task in which participants were not obliged to focus on a single time period and thus could report whichever type of event came first to mind. However, we did find a developmental shift, with adults (and to some extent adolescents) reporting that future events came to mind more rather than less quickly than past events. We note Coughlin et al.’s findings with adults were in the same direction. Finally, we found effects of time period on both clarity ratings (past events rated as more clearly envisaged than future events) and emotion ratings (future events were more positively rated than past events) that did not interact with age group. These findings are consistent with the existing adult literature which indicates that episodic memories tend to be reported as more vivid than imagined future events. With regard to emotion ratings, our findings provide new evidence that the future positivity bias is robust; it is present even in young children, and does not change in magnitude developmentally.

Finally, we consider the findings regarding the lack of relations between measures. Perhaps most surprisingly, we found no relation between the likelihood that participants produced future event descriptions in the word cue task and the likelihood that they made future-oriented reports when mind-wandering. In the mind-wandering literature, the tendency to produce future-oriented reports has been seen to be reflective of individual differences in working memory and has also been shown to be affected by mood and by depression (Baird et al., 2011; Hoffmann, Banzhaf, Kanske, Bermpohl, Singer, 2016; Smallwood et al., 2009; Smallwood & O’Connor, 2011). As we have already emphasized, we found no evidence of a relation between working memory and the tendency to produce future-oriented thoughts in either of our tasks. The lack of a relation between levels of future-oriented thinking across the two tasks suggests that future-oriented thinking may employ different processes across the two tasks, and moreover, that it may not make sense to talk about individual or group differences in the likelihood of engaging in future-oriented thought without specifying task context.

## Summary and conclusions

The aim of our study was to examine whether there were developmental changes in future-oriented bias. The findings do not straightforwardly support the idea that there are sizable developmental changes in such bias across the board. There were no developmental changes, even over a wide age-range, in the probability that participants produced a future rather than past event description in the word cue task, and similarly there were no developmental changes in the number of times that participants reported thinking about the future in the mind-wandering task. On these specific measures, there is no evidence of developmental change over even a very wide age-range. The lack of developmental change on the word cue task in particular suggests that the tendency to generate information about the past rather than the future in response to external cues is a very robust one that reflects a basic property of how information is retrieved from the systems involved in episodic thinking.

The lack of a developmental change in the number of times that participants report future thoughts in the mind-wandering task makes it difficult to argue that the future is not salient for younger age groups. Nevertheless, we did find some developmental changes in both our tasks. More than a third of our youngest groups never produced any future event descriptions in the word cue task, a significantly higher proportion than in any other group, indicating that some younger children find it particularly difficult to produce future event descriptions. Moreover, in the mind-wandering task, unlike with adults, a future-oriented bias (thinking more about the future relative to the past when mind-wandering) was not found in the child/adolescent groups. Thus, our findings suggest that in the context of spontaneously produced thoughts about other times, the future is less dominant than the past in the minds of children and adolescents than adults. Taken together, these findings indicate that there are some developmental changes in future thinking, and that even adolescents may not resemble adults in the degree to which they focus on the future relative to the past.
